# Differential Immune Response to Hydroxyapatite Precursors Under Inflammatory Pressure: In Vitro and In Vivo Studies

**DOI:** 10.3390/cells15020101

**Published:** 2026-01-06

**Authors:** Irina S. Fadeeva, Anastasia Yu. Teterina, Igor V. Smirnov, Vladislav V. Minaychev, Mikhail A. Shlykov, Margarita I. Kobyakova, Polina V. Smirnova, Anatoliy S. Senotov, Alena I. Zvyagina, Viktor A. Palikov, Arina V. Kholina, Eugeny S. Mikhaylov, Roman S. Fadeev, Vladimir S. Komlev

**Affiliations:** 1 Baikov Institute of Metallurgy and Materials Science, Russian Academy of Sciences, Leninskiy Prospect 49, Moscow 119334, Russia; teterina_imet@mail.ru (A.Y.T.); baldyriz@gmail.com (I.V.S.); vminaychev@gmail.com (V.V.M.); ceshakov@gmail.com (M.A.S.); kobyakovami@gmail.com (M.I.K.); smirnova-imet@mail.ru (P.V.S.); 2 Institute of Theoretical and Experimental Biophysics, Russian Academy of Sciences, Institutskaya Str. 3, Pushchino 142290, Russia; a.s.senotov@gmail.com (A.S.S.); alennazvyagina@gmail.com (A.I.Z.); fadeevrs@gmail.com (R.S.F.); 3 Branch of Shemyakin-Ovchinnikov Institute of Bioorganic Chemistry, Russian Academy of Sciences, Prospect Nauki 6, Pushchino 142290, Russia; vpalikov@bibch.ru (V.A.P.); bervinova@bibch.ru (A.V.K.); mikhaylov@bibch.ru (E.S.M.)

**Keywords:** osteoimmunology, low-temperature calcium phosphate, biocompatibility, immune cell, material-associated inflammation

## Abstract

The clinical success of calcium phosphate bone grafts (CPs) largely depends on the body’s immune response. However, traditional biocompatibility tests use healthy organisms and cannot predict effectiveness in patients with common chronic inflammatory diseases. This study examines how inflammation modulates the immune response, in vitro and in vivo, to low-temperature biomimetic CPs: dicalcium phosphate dihydrate (DCPD), octacalcium phosphate (OCP), and hydroxyapatite (HAp). In vitro studies involved human monocytes, macrophages, lymphocytes, and mesenchymal stromal cells (MSCs), with or without pro-inflammatory activation. In vivo biocompatibility was assessed via subcutaneous implantation in rats, with or without Complete Freund’s Adjuvant (CFA)-induced inflammation. Under normal conditions, all CP caused minimal immune reactivity. Inflammation-activated macrophages, however, triggered an acute reaction with significantly increased TNF-α and IL-1β secretion. Healthy and inflamed animals showed sharp contrasts. Although all materials exhibited thickened fibrous capsules during inflammation, biocompatibility varied markedly: DCPD performed best by promoting angiogenesis with minimal inflammation; HAp provoked the most severes response, including tissue necrosis and signs of rejection; OCP showed intermediate effects, with angiogenesis but notable fibrosis. Inflammatory processes critically influence CP biocompatibility; materials biocompatible in healthy organisms can induce fibrosis or rejection under inflammation. Disease-relevant, immune-challenged models are essential to predict clinical efficacy and safety.

## 1. Introduction

Bone defects from traumatic injuries, osteomyelitis, tumor resection, and prosthetic revisions pose major clinical challenges, requiring surgical bone grafts [1,2,3]. Implant surgery forms a significant portion of bone defect restoration procedures, where shortages of high-quality biomaterials remain a serious issue in traumatology, orthopedics, and tissue engineering [4,5].

Synthetic bone implants have long been commercially available; however, their use has largely been limited to maxillofacial surgery due to poor regeneration of critical-sized defects [6,7]. A new generation of calcium phosphate composites shows sufficient osteoinductivity for such defects [8,9,10], though bone repair (osteoreparation) and osseointegration processes remain incompletely understood [11].

Variability in biological effects ranges from full regeneration to recipient bone autolysis via osteoclast-mediated erosion from local inflammation [12]; this occurs across CaP materials and even among identical batches in patients with the same condition.

There is a growing focus in the scientific literature on the role of the recipient’s immune system in the process of osteogenesis [7]. Osteoimmunology posits immune responses as crucial for bone production and homeostasis [13]. The term “osteoimmunology” originated in a 2000 Nature editorial linking bone biology and immunology [14,15]. With proper osteoimmunomodulation, calcium phosphate materials acquire osteoinductive properties without the use of biologics such as recombinant proteins, gene therapies, or cellular components, thereby avoiding the more stringent requirements associated with combination products [16,17].

Inflammatory immune cells facilitate bone tissue regeneration and are involved in the subsequent processes of fracture healing [18,19]. Controlled local inflammation is crucial for complete bone regeneration; however, hyper-inflammatory conditions following major trauma are linked to impaired fracture healing [20].

A diverse range of immune cells plays a key role in the process of osteogenesis, including myeloid cells (neutrophils, monocytes, macrophages, and mononuclear giant cells) and lymphoid cells (T and B lymphocytes), as well as resident bone tissue cells—mesenchymal stromal cells, osteoblasts, and osteoclasts [21,22]. The heterogeneity of immune cells is not limited to traditional classifications and is manifested in the classical M1/M2 polarization of macrophages and the corresponding N1/N2 classification of neutrophils [23,24].

Upon fracture, neutrophils and monocytes are the first to migrate to the site of injury to prevent pathogen penetration [25,26]. Monocytes differentiate into macrophages, which, in the event of inflammation, adopt a pro-inflammatory M1 phenotype and commence the secretion of cytokines, including tumor necrosis factor-α (TNF-α), interleukin-6 (IL-6), and IL-1β [27]. Granulocytes, in turn, promote angiogenesis and hematopoiesis regeneration by delivering TNF-α to endothelial cells [28].

At the subsequent proliferative stage, in response to changes in the cellular and cytokine microenvironment, highly plastic macrophages predominantly switch to an anti-inflammatory and angiogenic M2 phenotype [29]. Infiltrating M2 macrophages, represented by the inflammation-suppressing and angiogenesis-stabilizing M2a phenotype, the immunoregulatory M2b phenotype, and the M2c phenotype associated with angiogenesis, matrix remodeling, and phagocytosis, activate the adaptive T-cell immune response. By secreting VEGF and immunomodulatory cytokines (IL-10 and TGF-β), they significantly contribute to bone tissue healing while adapting to changes in the microenvironment [12,30,31]. Furthermore, it has been shown that enhanced migration of mesenchymal stromal cells is mediated by macrophage-secreted MCP-1 and MIP-1α [32].

Osteoclasts, which originate from the same hematopoietic precursor as macrophages, are multi-nucleated cells capable of efficiently degrading both the organic and inorganic fractions of bone. Osteoclastogenesis is essentially regulated, both in vivo and in vitro, by macrophage colony-stimulating factor (M-CSF) and the tripartite system constituted by the receptor activator of nuclear factor κB (RANK), its ligand (RANKL), and osteoprotegerin (OPG). RANKL enhances dendritic cells’ capacity to induce naive T-cell proliferation, hence serving as a potential regulator of T-cell-dependent immune responses [33]. RANKL may additionally augment bone resorption in cases of humoral hypercalcemia [34]. Numerous pieces of evidence highlight the critical role of osteoclasts in the process of bone tissue regeneration [35,36,37].

Consequently, the body’s immune response to material implantation determines the success of bone repair. A material must be not only osteogenic (inducing the osteogenic differentiation of stem cells) but also osteoimmunomodulatory, i.e., it must interact with immune system cells and create a favorable microenvironment for osteogenesis. Many modern materials successfully accomplish this task [16,17].

It should be noted that most studies on biomaterials do not consider their biological properties in the context of interactions with the immune system. However, it is precisely immune cells that first come into contact with an implanted material and largely determine its subsequent biological response. It is known, for example, that hyperactivation of the immune system promotes osteoclastogenesis and shifts the balance of bone remodeling toward resorption [38,39]. Accordingly, the immune status of the patient is one of the key factors influencing the realization of the osteogenic properties of a material in the body. However, the effects of both normal and heightened immune activity, including chronic inflammatory conditions, on the biological properties of biomaterials remain insufficiently studied. This is especially relevant for bone substitute materials, which are widely used in elderly patients who often have comorbidities and chronically activated immune systems [40].

The objective of this work is to investigate the role of immune status in modulating the host response to biomaterials The goal was to answer the following question: how significantly does the response of cells and tissues to CP materials change in vitro and in vivo under normal conditions versus under inflammatory conditions?

## 2. Materials and Methods

The initial reagents were purchased from Sigma-Aldrich (Saint Louis, MO, USA) and used as received. All chemicals and solvents, except for those stated below, were purchased commercially and used without further purification unless otherwise stated.

### 2.1. Synthesis Procedure and Characterization

**Figure 1:**
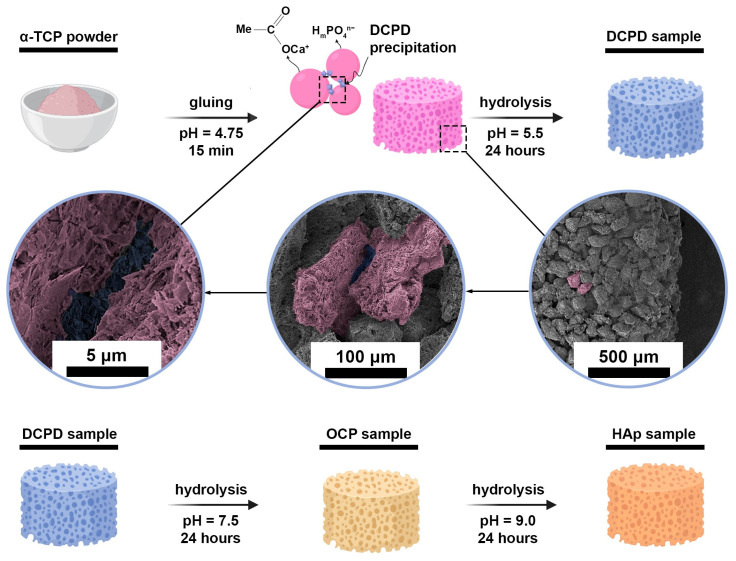
Graphical scheme of obtaining experimental three-dimensional samples of CPs.

For this study, cylindrical samples were prepared. They were obtained from tricalcium phosphate (TCP) powder, which was hydrolyzed in several stages to obtain CPs according to the following process: TCP → DCPD → OCP → HAp (Figure 1). These phases (except TCP) are thermodynamically unstable under organism conditions but kinetically favorable compared to HAp [41]. Therefore, they are intermediate phases during natural apatite crystallization in vivo and can be detected in bone tissue [42,43]. Materials based on these phases (OCP, DCPD) also undergo phase transformations in vivo, crystallizing in the form of HAp [44,45].

TCP powder was obtained by precipitation from an aqueous solution of salts: 0.5M (NH_4_)_2_HPO_4_ and 0.5M Ca(NO_3_)_2_∙4H_2_O, with a Ca/P ratio of 1.50 (21 °C, pH = 7.0 ± 0.1, maintained by dropwise addition of NH_4_OH(aq) (SigmaTek, Moscow, Russia)). After synthesis, the TCP powder was sintered at 1300 °C for 2 h, then it was ground in a mortar to obtain a particle size of 60–100 μm and pressed into cylindrical samples with geometric dimensions of 12.5 mm × 4.5 mm, which were immersed in an acetic acid solution with pH = 4.5 ± 0.1 at a volume of 0.5 mL per cylinder for 15 min to glue the particles. Cylinders were needed for normalized heterotopic (subcutaneous) implantation.

DCPD samples were obtained by keeping TCP cylinders in a 1.5 M sodium acetate and 0.15 M L-glutamic acid solution (pH of 5.5 ± 0.2, 35 ± 2 °C, 24 h).

A 1.5 M sodium acetate solution with a pH of 9.0 ± 0.2 (35 ± 2 °C, 24 h) was used to hydrolyze DCPD cylinders in order to obtain OCP samples.

HAp samples were obtained by hydrolysis of OCP cylinders in a 2.0 M sodium acetate solution (pH 9.7 ± 0.2, 40 ± 2 °C, 24 h).

Each hydrolysis step was carried out with a sample/solution weight ratio of 1/100, with constant stirring in a shaker-incubator. Drying of the samples was carried out in a thermostat at a temperature of 37 ± 1 °C.

The microstructure and morphology of tablet surfaces and slices were studied using a microscope Tescan VEGA III (scanning electron microscopy (SEM), Brno, Czech Republic). Prior to imaging, samples were covered with gold by a Q150R Quorum Technologies (Lewes, UK). Surface images of the materials were obtained at pressures of 7.3 × 10^−2^ Pa in the column and 1.5 × 10^−1^ Pa in the chamber.

X-ray diffraction (XRD) analysis was performed on the surface of the cylinders using an X-ray diffractometer “TD-3700”. Diffraction patterns were studied in the 2θ range of 4–55° at a tube voltage of 40 kV and a current of 40 mA. Full-profile Rietveld analysis was performed for all samples using Jana software (2006; Rwp < 0.15). The original crystal structures were obtained from the open-source database (COD) (No. 1533075, 7217893, and 9011091 for DCPD, OCP, and HAp, respectively).

On a Vertex 70v vacuum spectrometer (Bruker, Billerica, MA, USA) operating in the 400–4000 cm^−1^ range with a resolution of 4 cm^−1^, FTIR spectra were acquired from cylinder surfaces using attenuated total reflection Fourier IR spectroscopy (FTIR-ATR).

X-ray microtomography study (microCT) was carried out using a Bruker “SKYSCAN 1275” (Bruker microCT, Kontich, Belgium) at a resolution of 4.5 µm, providing a detailed analysis of porosity morphological, density, and material thickness using the Comprehensive TEX Archive Network (CT-an) program. Images were captured with 0.73° at each pace, with a voxel size of 13.76 µm, and further reconstructed using NRecon^TM^ v.1.6.8.0, SkyScan, 2011 (Bruker micro-CT, Kontich, Belgium). Ring artifact and beam hardening corrections were applied in reconstruction. Afterward, the reconstructed images were realigned using the Data Viewer TM 1.4.4.0 software (Bruker micro-CT, Kontich, Belgium).

### 2.2. In Vitro Studies

#### 2.2.1. Cell Lines

Human peripheral blood CD14+ monocytes (PBMs), human peripheral blood T cells, human peripheral blood B cells, and human bone marrow stromal cells (MSCs) were procured from CLS Cell Lines Service GmbH (Eppelheim, Germany). PBM, PBT, and PBB were cultivated in a medium specifically designed for human blood cells (CLS Cell Lines Service GmbH, Eppelheim, Germany) supplemented with gentamicin sulfate (40 μg/mL) at 37 °C in a humidified environment containing 5% CO_2_. Mesenchymal stem cells were grown in Human Marrow Stromal Cell Growth Medium (CLS Cell Lines Service GmbH, Eppelheim, Germany) supplemented with 40 μg/mL of gentamicin sulfate at 37 °C in a humidified environment containing 5% CO_2_.

Macrophages were obtained from peripheral blood mononuclear cells (PBMs). PBM were cultivated in a modified Eagle Medium supplemented with 10% fetal bovine serum (FBS) from Gibco in Waltham, MA, USA, and 40 μg/mL gentamicin sulfate at 37 °C in a humidified environment containing 5% CO_2_. The culture medium was substituted with a new medium three days post-cell seeding and subsequently with DMEM augmented with 2% FBS after an additional four days. Cells were cultured for seven days in low-serum medium prior to use in experiments. Cell dissociation was performed using Accutase solution to detach macrophages from the culture vessel surfaces.

The MycoFluor^TM^ Mycoplasma Detection Kit (Thermo Sci., Waltham, MA, USA) was used to examine cell cultures for mycoplasma infection. The cell cultures did not reveal any signs of mycoplasma infection.

#### 2.2.2. Cell Viability Assays

Cells were seeded in a 96-well plates (SPL Life Science, Pocheon, Republic of Korea) at a concentration of 1 × 10^4^ cells per well in 100 μL of medium and cultured for 24 h. Cells were then added to DCPD, OCP, and HAp samples at concentrations of 0.1, 0.3, 1, 3, and 10 mg/mL and co-cultured for another 96 h. CP samples were pre-sterilized with 75% ethanol according to the indicated method [46]. Cell viability was assessed by the flow cytometer BD Accuri C6 (BD Bioscience, Franklin Lakes, NJ, USA) after incubating cells with 200 nM Calcein AM fluorescent probe in culture medium.

#### 2.2.3. CD Markers Cell Surface Expression Analysis

To study the surface expression of CD markers, the cells were harvested from culture flasks and washed in cell-staining buffer at 300 g for 5 min. Staining was performed using monoclonal antibodies PE anti-human CD25 and APC anti-human CD71 (all from BioLegend, San Diego, CA, USA). To control for nonspecific binding, cells were incubated with isotype-matched control antibodies. Staining was performed at room temperature in the dark for 30 min. After staining, the cells were fixed with 2% paraformaldehyde solution, and CD surface expression was analyzed using a BD Accuri C6 flow cytometer (BD Bioscience, Franklin Lakes, NJ, USA) [47].

#### 2.2.4. Phagocytosis Activity

Phagocytic activity was assessed after a 2 h incubation of cells in growth medium containing 1 mg/mL pHrodo Green *E. coli* (Thermo Fisher Scientific, Waltham, MA, USA). To minimize nonspecific staining, cells were pre-treated with 10 μg/mL cytochalasin D for 30 min in a CO_2_ incubator prior to exposure to 1 mg/mL pHrodo Green *E. coli* for an additional 2 h [48]. The fluorescence was measured on an Infinite F200 PRO plate reader (Tecan, Männedorf, Switzerland) at an excitation wavelength of 485 nm and an emission wavelength of 530 nm. Phagocytic activity was quantified as the mean fluorescence intensity per cell in fluorescent cells, referred to as the phagocytic number. To simulate inflammation, cells were pre-incubated with 10 μg/mL LPS from *E. coli* O111: B4 for 24 h.

#### 2.2.5. LysoTracker Staining

To assess the acidic compartments in cells after 96 h of co-incubation with DCPD, OCP, and HAp, cells were washed three times with phosphate-buffered saline (PBS) and stained with 50 nM LysoTracker Green DND-26 (Thermo Fisher Scientific, Waltham, MA, USA) for 30 min in a CO_2_ incubator. Control cells were incubated with 50 μM chloroquine for 4 h [48]. Fluorescence was measured using an Infinite F200 PRO plate reader (Tecan, Männedorf, Switzerland) at an excitation wavelength of 485 nm and an emission wavelength of 530 nm.

#### 2.2.6. ROS Production Assay

To evaluate the production of reactive oxygen species (ROSs) in cells, after 96 h of co-incubation with DCPD, OCP, and HAp, cells were washed three times with PBS solution and stained with 20 μM 2′,7′-dichlorodihydrofluorescein diacetate (DCFH-DA) for 15 min in a CO_2_ incubator [49]. As a control, cells were incubated with 1 mM hydrogen peroxide for 20 min. Fluorescence measurements were analyzed using a BD Accuri C6 flow cytometer (BD Biosciences, Franklin Lakes, NJ, USA). A total of 3 × 10^4^ cells were analyzed for each sample.

#### 2.2.7. Intracellular Nitric Oxide (NO) Activity

Intracellular nitric oxide (NO) levels were assessed by incubating cells with 5 μM DAF-FM DA for 40 min in a CO_2_ incubator. Samples were subsequently rinsed with fresh medium and cultured for an additional 30 min in a CO_2_ incubator. To induce NO production, cells were incubated with 10 μg/mL LPS from *E. coli* O111:B4 for 24 h [45]. Cell fluorescence was assessed with a BD Accuri C6 flow cytometer (BD Biosciences, Franklin Lakes, NJ, USA).

#### 2.2.8. Cytokines Measurement by ELISA

Cell culture supernatants were collected by centrifugation (300× *g*, 5 min). TNF-α, IL-1β, IL-6, IL-13, and VEGF concentrations were determined using commercial ELISA kits (interleukin-1beta-ELISA-BEST, interleukin-6-ELISA-BEST, and alpha-TNF-ELISA-BEST from Vector-Best Inc., Novosibirsk, Russia; IL-13 and VEGF kits from Cloud-Clone Corp., Wuhan, China) according to the manufacturer’s instructions. Absorbance was measured at 450 nm using an iMark microplate reader (Bio-Rad, Hercules, CA, USA). Cytokine levels were normalized to 5 × 10^4^ cells/mL.

### 2.3. In Vivo Studies

#### 2.3.1. Animals

The study was conducted at the Biological Testing Laboratory of the BIBCh RAS, accredited by the Association for Assessment and Accreditation of Laboratory Animal Care International (AAALAC), in accordance with the standards of the Guidelines for the Care and Use of Laboratory Animals (8th ed., Institute of Laboratory Animal Research). Outbred SPF male Wistar rats aged 7–8 weeks (190–215 g) at the beginning of the experimental procedures were obtained from the Nursery of Laboratory Animals “Pushchino” in the Branch of Shemyakin–Ovchinnikov Institute of Bioorganic Chemistry Russian Academy of Sciences (BIBCh RAS), Russian Academy of Sciences (Pushchino). All animal manipulations complied with the International Principles of the World Health Organization Guidelines on Biomedical Research involving Animals and were reviewed and approved by the Institute’s Commission on Humane Treatment of Animals (IACUC Protocol number: 898/22, approval date: 8 December 2022). Animals were acclimatized for two weeks in barrier rooms under controlled conditions: temperature 20–24 °C, relative humidity 30–60%, and a 12 h light/dark cycle. They received ad libitum access to standard rodent diet (Mucedola Standard Diet 4RF21, Settimo Milanese, Italy) and filtered water (Milli-Q HR 7060 Water Purification System, Merck KGaA, Darmstadt, Germany). Cages were bedded with dust-free wood chips (LIGNOCEL BK8/15, JRS, Rosenberg, Germany) and enriched with polycarbonate shelters (Techniplast S.p.A., Buguggiate, Italy).

#### 2.3.2. Complete Freund’s Adjuvant (CFA)-Induced Inflammation

Animals displaying no signs of health abnormalities were selected for the experiment following clinical examination. The animals were randomly allocated into eight experimental groups (*n* = 5 per group). Randomization was performed using body weight as the primary criterion to ensure that the mean body weight across all groups did not differ statistically on the first day of the study.

Four groups (1–4) served as non-sensitized native controls. In the remaining four groups (5–8), inflammation was induced via the Complete Freund’s Adjuvant (CFA) protocol. To induce paw inflammation, CFA (Sigma-Aldrich, Saint Louis, MO, USA) was suspended in a 1:1 oil/saline emulsion. Inflammation was induced by injecting 100 µL of CFA emulsion into the plantar surface of the left hind paw in experimental rats; control animals received 100 µL of saline. After seven days, rats were subcutaneously implanted with calcium phosphate (CP) materials (2 mg/kg) or saline for controls (Table A1).

#### 2.3.3. Surgical Implantation of CPs

A model of ectopic implantation of experimental biomaterials was used to study the biocompatibility and blood immune response of the obtained CPs in vivo (ISO 10993. Part 6. Tests for local effects after implantation [50]). All surgical procedures, handling, and housing were approved by the Committee for Control over Care and Use of Laboratory Animals of BIBCh RAS (IACUC). Surgical procedures were performed according to previously established protocols [49]. Briefly, 42 Wistar rats (~180 g) (Pushchino nursery of laboratory animals, Pushchino, MO, Russia) were used. General anesthesia was achieved using a combination of xylazine (13 mg/kg; Interchemie, Venray, The Netherlands) and zoletil-100 (7 mg/kg; Virbac, Carros, France). Before implantation, samples were sterilized by soaking in 70% ethanol for 2 h and drying in a sterile box under a UV lamp. The samples were implanted subcutaneously in the dorsal region of the rats. Animals from each group were divided to be euthanized (carbon dioxide protocol) after four weeks (twenty-eight days) of the experiment.

#### 2.3.4. Hematological Analysis

Hematological analysis was performed on the 7th, 17th, and 28th day of the experiment. Blood was sampled through the caudal vein. Whole-blood analysis was conducted using EDTA K-3 tubes within 2–3 h after obtaining a sample for all parameters using a Mythic 18 Vet hematology analyzer (C2 Diagnostics, Montpellier, France).

#### 2.3.5. Histological Analysis

The CP cylinders were explanted with the surrounding tissue and assessed as described below for subsequent morphological study. Immediately after humane euthanasia, to prevent autolysis instantly after the withdrawal, samples of implanted materials with surrounding tissues of the recipient bed were washed for 30 s with a cold (14 °C) isotonic solution and fixed for 48 h in neutral buffered formalin (NBF) at a tissue-to-fixator volume ratio of 1:30. For morphological study, after fixation, samples of connective tissue capsules were dehydrated and paraffin-embedded. Sections (with a thickness of 4 μm) were prepared and stained with hematoxylin–eosin. Micrographs of the stained histological samples were obtained on a Nikon Eclipse Ti-E microscope station (Nikon, Tokyo, Japan) and processed using the NIS Elements AR4.13.05 software (Build 933). Scar tissue maturity around the samples was evaluated based on its thickness and the relative area of blood vessels. High maturity corresponds to minimal inflammation in low-thickness tissues with a small number of vessels.

### 2.4. Statistical Analysis

In vitro results are presented as mean ± standard deviation (M ± SD). Each in vitro and in vivo experiment was performed at least five times (*n* ≥ 5). Data were analyzed using Python 3 (version 3.10.6) with the following libraries: Pandas (1.5.3), NumPy (1.23.5), and SciPy (1.10.0) in the Spyder (version 5.4.1) integrated development environment.

The Mann–Whitney U test was used to compare two independent samples. Before conducting multiple comparisons between groups, data normality and homogeneity of variance were assessed using the Shapiro–Wilk and Levene tests, respectively, with SciPy. For non-normally distributed data, the Kruskal–Wallis H test was used, followed by comparison of the experimental groups using Tukey’s test. For normally distributed data, one-way analysis of variance (ANOVA) was used, followed by comparison of the experimental groups using Holm–Sidak test.

Graphical analysis and visualization were performed using Python 3.10.6 with Matplotlib (version 3.7.0) and Seaborn (version 0.12.2).

## 3. Results

### 3.1. Materials Characterization

**Figure 2:**
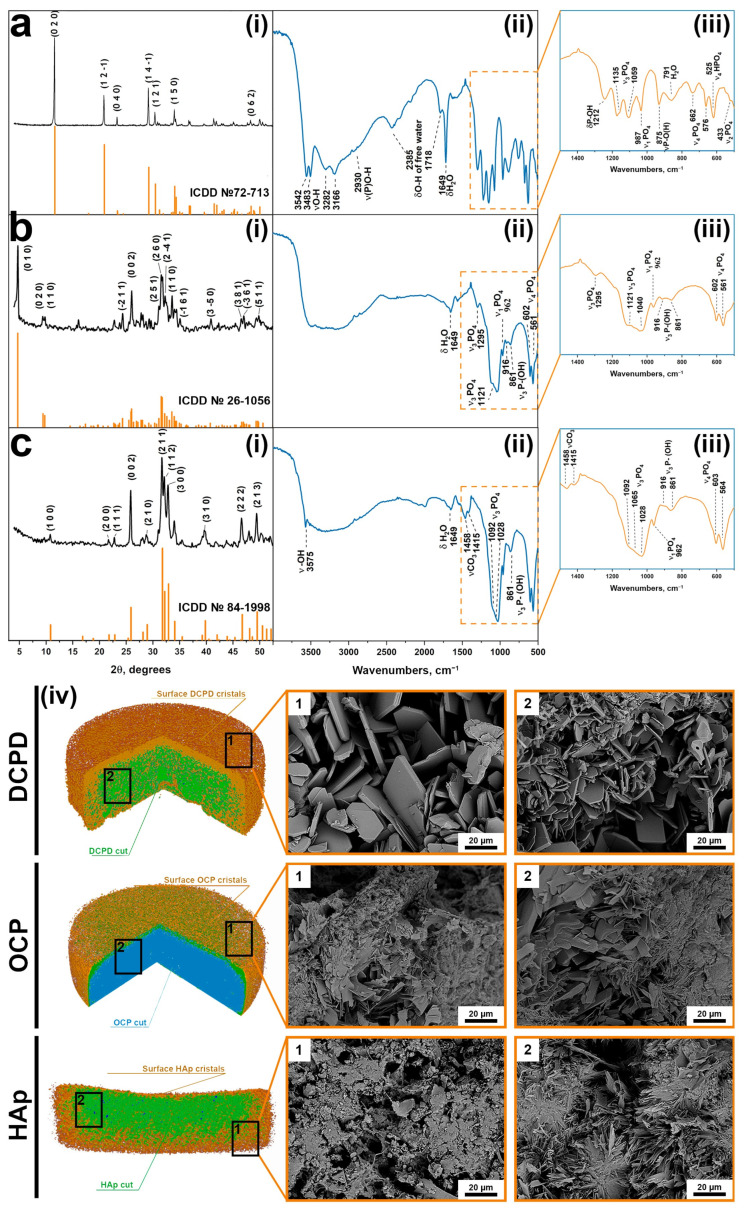
XRD patterns (**i**), FTIP spectra (**ii**,**iii**), and macro- (CT)/microstructure (SEM) (**iv**) of the cylinders: (**a**) DCPD; (**b**) OCP; (**c**) HAp.

XRD analysis was performed on the surface of the cylinders, and the results are shown in Figure 2(i). The DCPD, OCP, and HAp spectra correspond to cards No. 72-713, 26-1056, and 84-1998 of the XRD base ICDD (Powder Diffraction File, Alphabetical Index Inorganic Compounds, Pennsylvania: JCPDS, 1997). Whole-blood analysis was conducted using EDTA K-3 tubes within 2–3 h after obtaining a sample for all parameters using a Mythic 18 Vet hematology analyzer (C2 Diagnostics, Montpellier, France). 

FTIR analysis was performed on the surface of the cylinders, and the results are shown in Figure 2(ii,iii) [50]. The DCPD spectrum has characteristic modes of the HPO_4_^2−^ group at 433 cm^−1^ (ν2), at 987, 1059, and 1135 cm^−1^ (ν3), and at 525, 576, and 662 cm^−1^ (ν4). O-H stretching of lattice water occur at 3542, 3483, 3282, and 3166 cm^−1^. After phase transformation to OCP, the O-H stretching modes turned into a plateau at 3000–3500 cm^−1^. New characteristic modes assigned to PO4^3−^ groups appear, including bending motions (ν4) at 561 and 602 cm^−1^ and P-O stretching modes at 962 cm^−1^ (ν1) and at 1140 and 1121 cm^−1^ (ν3).

Similar to the results of XRD, FTIR confirms the transformation of OCP into HAp. A sharp and narrow band at 3566 cm^−l^ belongs to the internal stretching of the OH^−^ ion. This conversion is also marked by a relative decrease in peaks at 861, 917, 1103, 1121, and 1295 cm^−1^, which are attributed to the HPO_4_^2−^ group. The stretching band of absorbed water occurs at 1649 cm^−1^. Bands at 1415 and 1458 cm^−1^ are assigned to the stretching vibrations of the CO_3_^2−^ group.

At the macro level, according to microCT data, it can be seen that the three-dimensional samples are cylindrical (Table A2), with a pronounced surface relief (Figure 2(iv)). Microporosity is present in all samples.

The microstructure of the samples underwent significant changes during the DCPD → OCP → HAp phase transformation sequence (Figure 2(iv)). Scanning electron microscopy analysis revealed that DCPD samples initially consisted of plate-shaped crystals with lateral dimensions of 30–50 μm and a thickness of 2–5 μm. This morphology is attributed to differential crystal growth activity on the surface compared to the interior of the samples. Upon transformation to OCP, the surface of the samples became covered with newly formed plate-shaped OCP crystals with sizes up to 10 μm and a reduced thickness of approximately 1 μm. The internal structure of the materials became denser due to the inherent nature of the transformation process, although discrete clusters of crystals comprising individual units up to 5 μm in size were still observable. Subsequent transformation from OCP to HAp involved extensive dissolution of the OCP phase and concomitant precipitation of Hap, resulting in a marked reduction in surface crystal size to about 1 μm and a pronounced densification of the internal structure, where discrete HAp crystals were no longer distinguishable.

### 3.2. Results of In Vitro Analyses

**Figure 3:**
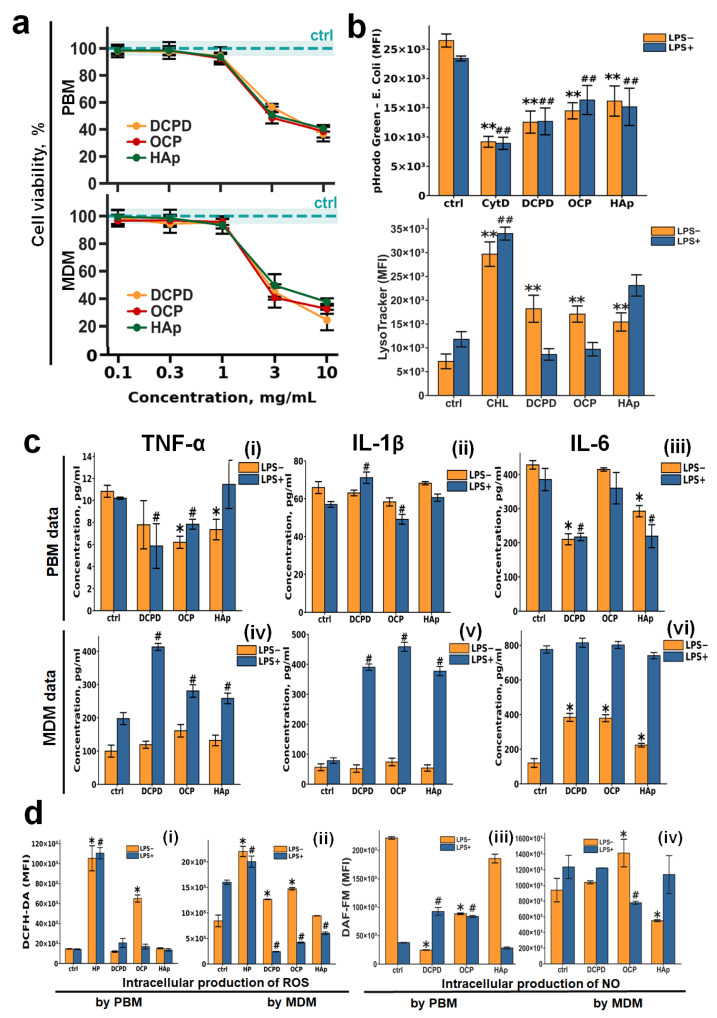
The effects of DCPD, OCP, or HAp on peripheral blood monocytes (PBMs) and monocyte-derived macrophages (MDMs). (**a**) Cell viability in human peripheral blood monocytes (PBMs) and monocyte-derived macrophages (MDMs) was assessed following 72 h incubation with DCPD, OCP, or HAp (**b**) *E. coli*—phRhodo Green conjugates and LysoTracker Green fluorescence (MFI) in MDM after 72 h of co-incubation with CPs. (**c**) Constitutive and LPS–induced secretion of TNF-α, IL-1β, and IL-6 by PBM and MDM after 72 h of incubation with DCPD, OCP, and HAp; the concentration of cytokines was normalized to 5 × 10^4^ cells/mL. (**d**) Constitutive and LPS–induced production of reactive oxygen species (ROSs) (**i**,**ii**) and nitric oxide (NO) (**iii**,**iv**) by PBM (**i**,**iii**) and MDM (**ii**,**iv**) after 72 h of incubation with DCPD, OCP, and HAp. LPS—lipopolysaccharide; CoA—concanavalin A; PWM—pokeweed mitogen; Ctrl—untreated cells without addition of CP materials; CHL—chloroquine; CytD—cytochalasin D; LPS—lipopolysaccharide; MFI—mean fluoresce intensity; HP—hydrogen peroxide. Results are expressed as mean ± standard deviation (*n* = 5). Statistical significance was determined using the Mann–Whitney U test: asterisks (*) indicate *p* < 0.05 and (**) indicate *p* < 0.01 compared to LPS− controls; pound symbols (#) indicate *p* < 0.05 and (##) indicate *p* < 0.01 compared to LPS+ controls.

**Figure 4:**
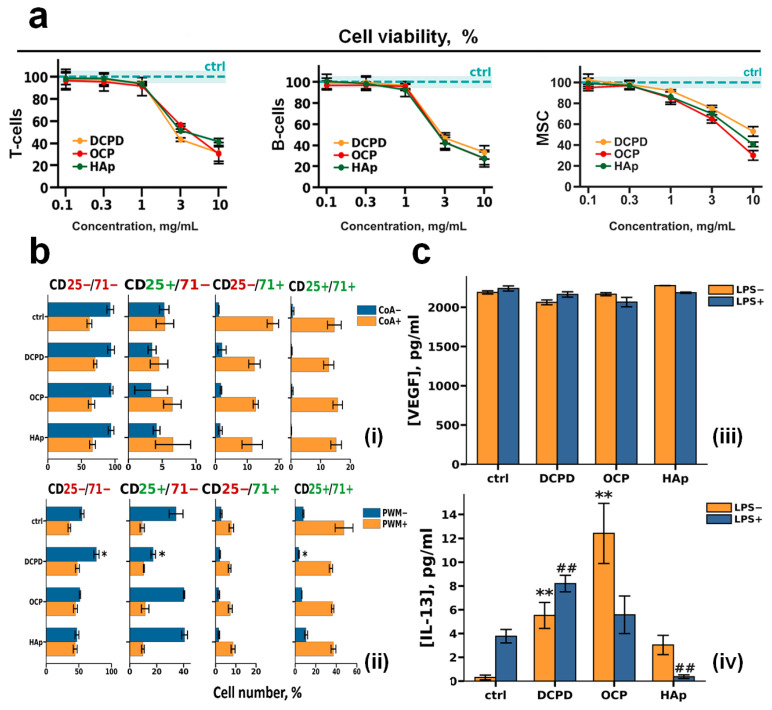
The effects of DCPD, OCP, or HAp on human peripheral blood T cells and B cells and human bone marrow stromal cells. (**a**) Viability of T cells, B cells, and bone marrow stromal cells (MSCs) after 72 h of co-incubation with DCPD, OCP, or HAp. (**b**) Analysis of the number of T cells (**i**) and B cells (**ii**) positive for CD25 and CD71 (CD25+/CD71+), only CD25 (CD25+/CD71−), only CD71 (CD25−/CD71+), or negative for both CD25 and CD71 (CD25−/CD71−). (**c**) Constitutive and LPS–induced secretion of VEGF (**iii**) and IL-13 (**iv**) by MSC after 72 h of cultivation with DCPD, OCP, and HAp; the concentration of cytokines was normalized to 5 × 10^4^ cells/mL. LPS—lipopolysaccharide; CoA—concanavalin A; PWM—pokeweed mitogen. Results are expressed as mean ± standard deviation (*n* = 5). Statistical significance was determined using the Mann–Whitney U test: asterisks (*) indicate *p* < 0.05 and (**) indicate *p* < 0.01 compared to LPS− controls; pound symbols (##) indicate *p* < 0.01 compared to LPS+ controls.

It was shown that DCPD, OCP, and HAp samples have no toxicity at concentrations of 1 mg/mL or lower for all cell types (Figure 3a and Figure 4a). To further study the effects of DCPD, OCP, and HAp on monocytes and MDM, this concentration (1 mg/mL) was used. The effects of CP samples on monocytes and macrophages under pro-inflammatory conditions were studied. For this purpose, PBM and MDM were treated with 10 µg/mL LPS for 24 h. Treatment of macrophages with lipopolysaccharide (LPS) is a classical model for innate immune activation. LPS, a major component of the outer membrane of Gram-negative bacteria, triggers a cascade of powerful reactions within the phagocytes.

#### 3.2.1. Phagocytic Activity of Macrophages and Monocytes

DCPD, OCP, and HAp (1 mg/mL) were assessed for their effects on phagocytic activity and acidic cellular compartments, including lysosomes, in macrophages (Figure 3b). The fluorescence intensity of the pHrodo Green dye (*E. coli* conjugate) decreased upon incubation with CPs under both normal (LPS−) and inflammatory conditions (LPS+) (Figure 3b). Concurrently, LysoTracker Green fluorescence intensity increased under normal conditions (LPS−) and remained unchanged under inflammatory conditions (LPS+) when monocytes were incubated with CPs. This finding aligns with our previous results obtained using THP-1PMA macrophage-like cells [48].

#### 3.2.2. Cytokine Production by Macrophages and Monocytes

Given that inflammation is a crucial phase of tissue repair [45,46,47], we examined the influence of CP samples on the secretion of essential inflammatory mediators (TNF-α, IL-1β, IL-6), ROS, and NO by macrophages and monocytes, which are vital for the biointegration of bone implants.

Incubation of monocytes with calcium phosphates slightly reduced the secretion of TNF-α and IL-6, with this effect being most pronounced for DCPD; IL-1β secretion remained unchanged. No differences were observed between normal (LPS−) and inflammatory (LPS+) conditions.

When macrophages were incubated with calcium phosphate samples under normal conditions (LPS−), the production of IL-6 was slightly increased for all CPs, while TNF-α and IL-1β production remained unaltered. However, under inflammatory conditions (LPS+), CPs induced a sharp release of TNF-α and IL-1β cytokines and did not affect IL-6 production, with no differences detected among the specific CP types.

The production of reactive oxygen species by monocytes did not depend on either the type of CP or the conditions (LPS− or LPS+) (Figure 3d(i)). ROS production by macrophages was largely unchanged upon incubation with CPs under normal conditions (LPS−); however, under inflammatory conditions (LPS+), CPs significantly inhibited this process (Figure 3d(ii)).

Nitric oxide production by unstimulated (LPS−) monocytes decreased upon incubation with all CPs, with the effect being most notable for DCPD. However, upon LPS stimulation (LPS+) of monocytes, DCPD and OCP conversely increased NO production (Figure 3d(iii)).

Nitric oxide production by macrophages was largely independent of both the presence of CPs and the conditions (LPS− or LPS+) (Figure 3d(iv)).

#### 3.2.3. Interaction of CPs and Lymphocytes

**Figure 5:**
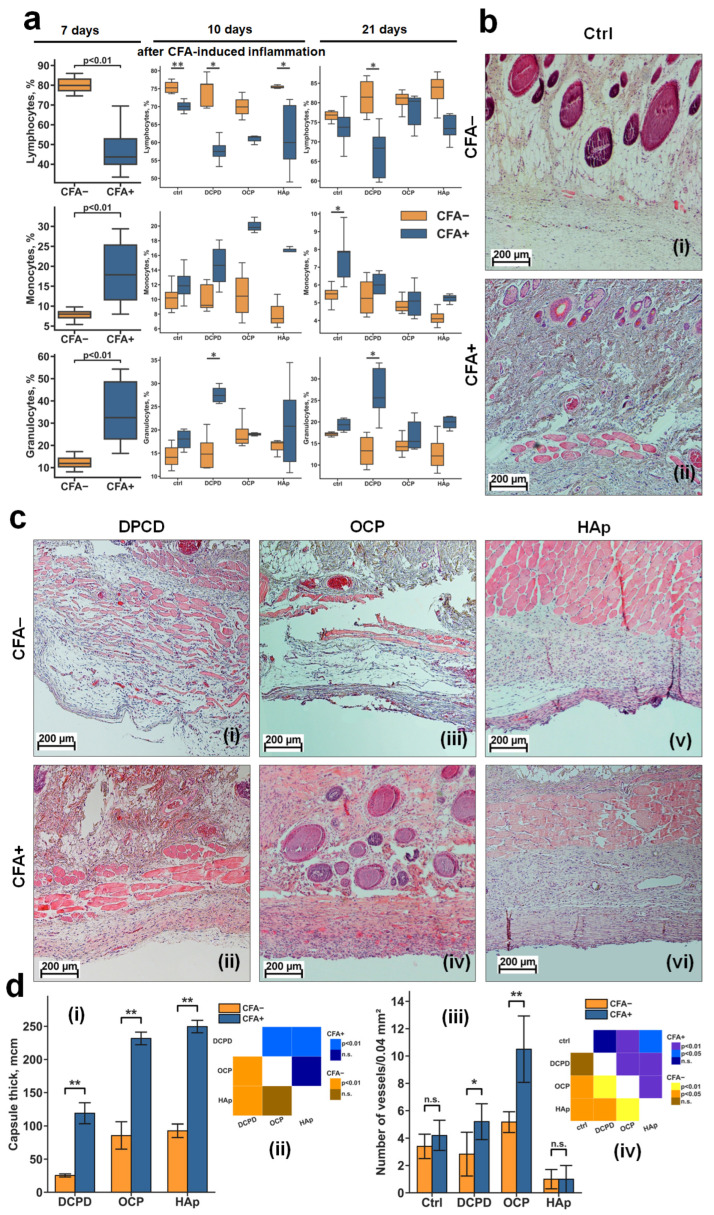
Relative content of blood cells and results of histological analysis of DCPD, OCP, and HAp samples in CFA− and CFA+ rats. (**a**) Relative content of blood cells in CFA− and CFA+ rats after 7, 17, and 21 days of CFA-induced inflammation, shown as boxplots with median, 25th and 75th quartiles, maximum and minimum values (*n* = 5). Asterisks indicate significant differences (* *p* < 0.05; ** *p* < 0.01 (Mann–Whitney U test)). (**b**) Light microscopy of subcutaneous tissues of CFA− (**i**) and CFA+ (**ii**) rats before implantation (day 7 of the experiment). (**c**) Light microscopy of fibrous capsule and peri-implant tissues with samples obtained after implantation of DCPD (**i**,**ii**), OCP (**iii**,**iv**), and HAp (**v**,**vi**) samples in CFA− (**i**,**iii**,**v**) and CFA+ (**ii**,**iv**,**vi**) rats (day 28 of experiment): hematoxylin–eosin staining: the nuclei of the cells are dark blue, the cytoplasm is light pink, and the matrix components are pink-purple, red blood cells are scarlet. (**d**) Fibrous capsule thickness (**i**) and number of blood vessels in capsules (**iii**) around DCPD, OCP, and HAp samples, and statistical significance matrices (**ii**,**iv**) of the data; data are shown as mean ± SD (*n* = 5); Ctrl: CFA− and CFA+ rats without implantation of CPs; (**i**,**iii**) n.s.—no significance; * *p* < 0.05; ** *p* < 0.01 (Mann–Whitney U test); (**ii**,**iv**)—statistical significance matrix (Kruskal–Wallis H test was used, followed by comparison of the experimental groups using Tukey’s test).

The effects of CP samples in concentrations of 1 mg/mL on lectin-dependent activation of human peripheral blood T and B cells were studied (Figure 4b). Lymphocyte activation was assessed by the surface expression of interleukin-2 receptor alpha chain (CD25) and transferrin receptor protein 1 (CD71), which are markers of cellular activation, after 72 h of treatment with 10 μg/mL concanavalin A (CoA) for T cells (Figure 4b(i)) and 5 μg/mL pokeweed mitogen (PWM) for B cells (Figure 4b(ii)) [51,52,53].

Our results showed that during incubation of both CoA-treated and untreated T cells with DCPD, OCP, and HAp, there was no change in the number of CD25+/CD71+, CD25−/CD71−, CD25+/CD71−, and CD25−/CD71+ T cells relative to control cells (without incubation with CPs) (Figure 4b(i)).

We also showed that the number of CD25+/CD71+, CD25+/CD71−, CD25−/CD71+, and CD25−/CD71− PWM-untreated B cells did not change in comparison with control cells during co-incubation with OCP and HAp. However, during incubation of PWM-untreated B cells with DCPD, the number of CD25+/CD71− and CD25+/CD71+ cells decreased, and the number of CD25−/CD71− cells increased compared with the control (*p* < 0.05 and *p* < 0.05, respectively). In turn, the number of CD25+/CD71+, CD25+/CD71−, CD25−/CD71+, and CD25−/CD71− cells did not change in comparison with the control cells during co-incubation of PWM-treated B cells with all CPs (Figure 4b(ii)).

#### 3.2.4. Interaction of CPs and Mesenchymal Stem Cells

To evaluate the impact of CPs on viability, morphology, and functional features of bone tissue cells, DCPD, OCP, and HAp samples (1 mg/mL) were incubated with mesenchymal stromal cells (MSCs) in normal (LPS−) and pro-inflammatory conditions (LPS+).

VEGF secretion remained consistent after 72 h of incubation with CPs samples of both LPS-treated (LPS−) and untreated (LPS+) MSC (Figure 4c).

After 72 h of incubation with DCPD, OCP, and HAp samples, the following dynamics of IL-13 secretion by mesenchymal stromal cells were observed: without LPS treatment, all three CPs induced de novo IL-13 secretion, with the most pronounced effect observed for OCP. With LPS+ stimulation, DCPD increased IL-13 levels, OCP did not affect the secretion level, and HAp suppressed IL-13 secretion.

### 3.3. Results of In Vivo Studies

#### 3.3.1. Histology Before Implantation

An experiment on subcutaneous implantation of CPs cylinders was conducted on healthy rats and rats with induced inflammation. Half of the rats were injected with 0.5 mg/mL CFA. On day 7, control samples were collected from CFA-treated (CFA+) and CFA-untreated (CFA−) rats, and the remaining rats were implanted with CP cylinders. Hematological analysis seven days after injection of 0.5 mg/mL CFA showed a significant change in the content of lymphocytes, monocytes, and granulocytes in experimental animals. CFA-treated (CFA+) rats showed a significant increase in the number of monocytes and granulocytes and decrease in lymphocytes compared with control CFA-untreated (CFA−) rats (Figure 5a).

Ten days after implantation of the CP materials (day 17 of the experiment), a significant decrease in lymphocyte content was observed between CFA+ and control CFA− rats for all groups. Relative to other cells, a significant difference between CFA+ and control CFA− rats was found only in the DCPD group, where granulocytes were increased in CFA+ rats (Figure 5a).

Twenty-one days after implantation (day 28 of the experiment), among all groups, significant differences between CFA+ and control CFA− rats were observed only in the DCPD group, where the CFA+ rats showed a decrease in lymphocytes and an increase in granulocytes (Figure 5a). The absence of significant differences in monocyte levels between CFA− and CFA+ groups on day 21 indicates attenuation of the acute systemic inflammatory response, suggesting that immune activity is now primarily localized to the implantation site.

Histological analysis of subcutaneous tissue of control CFA− rats at day 7 showed no signs of abnormality (Figure 5b(i)). In turn, in CFA+ rats, reactive changes in subcutaneous tissue and increased cell density were observed, indicating the formation of inflammatory conditions (Figure 5b(ii)).

Histological analysis of fibrous capsules and surrounding tissues 21 days after implantation (day 28 of the experiment) showed a difference in the response to implanted materials in both the control CFA− rats (Figure 5b) and CFA+ rats, and between the materials themselves within each group (Figure 5c).

#### 3.3.2. Rats Without Induced Inflammation (CFA−)

DCPD appeared to be the most biocompatible material. The fibrous capsule around the DCPD specimens was thin and friable, composed of mature collagen fibers saturated with fibroblasts, with no evidence of tight interlocking between them, and with an even content of a small number of mature blood vessels without inflammatory leukocyte infiltration (Figure 5b(i)).

The OCP material was less biocompatible and had a weak toxic effect but had a pronounced angiogenic effect. The capsule around these sample was much thicker and denser but contained many mature blood vessels (Figure 5c(iii)).

HAp was the least favorably perceived by the organism of the experimental animals. HAp samples demonstrated the thickest, occasionally multilayered fibrous capsule in the area of contact with the material, consisting of fused mature collagen fibers, predominantly containing leukocytes. HAp caused a reactive change in the dermal tissues, with an increase in cell density in the surrounding tissue, indicating the formation of inflammation in the contact tissues (compare Figure 5b(ii),c(v)).

#### 3.3.3. Rats with Induced Inflammation (CFA+)

DCPD retained signs of biocompatibility. The formation of reactively altered connective tissue was observed in the area of contact with the material, consisting of loosely fused collagen fibers containing mainly fibroblasts and leukocytes (Figure 5c(ii)). This capsule contained many small capillaries without signs of mature blood vessel formation, indicating delayed onset of capsule involution and signs of biocompatibility of this material in CFA+ rats. Although the peri-implant tissues were also altered, they were not visually different from those in CFA+ rats without implanted CPs materials. In general, despite the formation of reactively altered connective tissue around the materials, no evidence of leukocytes infiltration or dead cells was observed for DCPD samples.

For OCP samples, a relatively more pronounced negative reaction was observed—fibrous tissue overgrowth throughout the entire zone of contact with the implanted sample, with minimal fibroblast presence (indicative of secondary cell death within the fibrous capsule), and was predominantly populated by immune cells, suggesting significant material-associated local inflammation (Figure 5c(iv)). However, the strongest angiogenic effect was observed for OCP samples.

HAp samples had a pronounced inflammatory response. In certain samples, it was evidenced by epidermal thinning across the material’s surface, suggesting that a fistula may develop at this location in a subsequent implantation phase, accompanied by further material rejection. The majority of CFA + HAp samples exhibited development of a dense, acellular fibrous capsule characterized by collagen fiber fusion and indications of cellular death within the capsule, alongside a marked escalation in reactive alterations in the dermis and subcutaneous adipose tissue, signifying enhancement in CFA-induced inflammation (Figure 5c(vi)). HAp contained a significant amount of cellular debris at the border with surrounding tissues. Additionally, a notable presence of mast cells and histiocytes was observed. These findings suggest that the material lacks the requisite biocompatibility, despite the absence of inflammation.

Under inflammatory conditions (CFA+), DCPD demonstrated the best biocompatibility, while HAp demonstrated the worst, exacerbating the inflammatory response. OCP occupied an intermediate position, demonstrating both negative (fibrosis, inflammation) and positive (angiogenesis) effects.

The results have shown that, under induced inflammation, all materials significantly increased the thickness of the fibrous capsule around the implant (Figure 5d(i,ii)). This suggests that inflammation may trigger fibrosis instead of tissue regeneration. Moreover, under inflammatory conditions, DCPD and OCP materials, unlike HAp, were more effective in stimulating the formation of mature blood vessels at the tissue interface, particularly in an inflammatory microenvironment.

## 4. Discussion

This study involved the synthesis of low-temperature biomimetic CP materials (HAp and its precursors) and evaluation of their physicochemical properties. In vitro and in vivo research was conducted to examine the influence of biomimetic materials on essential cellular components of the immune system, particularly under induced inflammation in experimental animals. The biocompatibility and bioactivity of bone grafts, along with the overall health of the recipient’s immune system, significantly impact material-associated bone tissue repair.

The described synthesis method enables the reproducible production of low-temperature calcium phosphates in the form of cylindrical samples, which are convenient for subcutaneous implantation. The resulting samples are monophasic and highly crystalline.

In this work, we investigated the features of the immune cell response to various hydroxyapatite precursors in vitro, and their biocompatibility in a CFA-induced rat inflammatory model.

According to the World Health Organization, 20% of the global population suffers from allergies, 14% from diabetes, etc., and these numbers continue to rise [40]. Therefore, it is critically important to understand the immune response that bone implants elicit under conditions of altered immune status in the recipient.

Monocytes and macrophages play a central role in the response of recipient’s tissues to biomaterials [21,24]. During implantation of bone grafts, monocytes are the first cells to reach the implant surface. Subsequently, phagocytosis enables intracellular immune monitoring: degradation products of pathogens or materials are recognized by receptors (TLR, NLR), which, through the activation of signaling pathways (NF-κB, inflammasomes), determine the polarization of the subsequent cytokine response. The nature of this response depends on the properties of the phagocytosed object—its immunogenicity and resistance to lysosomal degradation [54,55].

In our study, both macrophages and monocytes phagocytosed CP particles. However, pHrodo Green enters the cell only through phagocytosis alongside CP particles and fluoresces at low pH. The decrease in MFI of pHrodo Green-stained cells indicates a rise in phagolysosomal pH due to CP dissolution, which is consistent with data on CP solubility [50]. In contrast, LysoTracker penetrates the cell membrane and, due to its chemical properties, accumulates in compartments with acidic pH, i.e., lysosomes and phagolysosomes. The increased LysoTracker MFI may indicate a greater number of acidic compartments in the cells. This is most likely a consequence of lysosomal stress—impaired phagocytosis triggers compensatory mechanisms (regulated by transcription factor EB), increasing the number of lysosomes, enzymatic activity, etc. It is possible that the direction of macrophage polarization and cytokine production is determined by the extent of lysosomal stress development [56].

In the case of monocytes without exposure to CPs, no significant differences in pro-inflammatory cytokine production were observed between control cells under normal and inflammatory conditions. This is likely due to the fact that monocytes, compared to macrophages, generally produce lower amounts of cytokines because of their circulating “resting” state and less developed signaling and secretory machinery, as well as their limited response even to a pro-inflammatory stimulus such as LPS [57,58,59].

However, this effect is clear for macrophages: under inflammatory conditions, their production of pro-inflammatory cytokines increases significantly, and the addition of CPs further enhances the production of IL-1β and TNF-α. Thus, it is apparent that the relatively neutral response of macrophages to calcium phosphate materials becomes sharply negative in the presence of inflammatory reactions in the body.

In addition to innate immunity (myeloid cells, in particular monocytes and macrophages), adaptive immune cells such as T cells and B cells play a significant role in the development of the immune response [60,61]. Lymphocyte activation is necessary for the formation of the “correct” reaction of the body in response to damage-associated molecular patterns (DAMPs) and pathogen-associated molecular patterns (PAMPs) [62,63]. In addition, when activated, T cells can express a nuclear factor ligand receptor activator (RANKL) on their surface to stimulate osteoclast maturation and bone resorption [64,65], as well as secrete humoral factors that may participate in suppressing osteogenic differentiation of mesenchymal stromal cells (MSCs). So, it is important to understand how CPs affect lymphocyte activation.

The data obtained showed that the DCPD, OCP, and HAp samples studied in this work did not affect the lectin-dependent activation of both human T cells and B cells during co-incubation in vitro. However, activation of adaptive immune response cells occurs in lymph nodes during antigen presentation, so the results of in vivo testing may vary.

MSCs and osteogenic cells of the bone marrow can take part in regulation of polarization, phagocytosis, and intracellular metabolism of phagocytes through secretion of several factors, such as IL-13 and vascular endothelial growth factor (VEGF) [66]. IL-13, an anti-inflammatory cytokine, can inhibit the secretion of pro-inflammatory mediators including nitric oxide (NO), IL-1β, IL-6, IL-12, and TNF-α [53,63,67]. In addition, IL-13 has been shown to suppress inflammatory cell infiltration and effectively trigger the transition of macrophages from the M1 to M2 state [68,69]. VEGF not only regulates bone homeostasis by interacting with certain humoral factors, but also interacts with osteogenic proteins such as BMP-2 to promote bone formation and bone healing by enhancing cell recruitment, prolonging cell survival, and enhancing angiogenesis [70,71].

The impact of CP materials on MSCs further underscores the significance of the inflammatory microenvironment. Our in vitro data revealed that under normal conditions, all tested CPs (DCPD, OCP, HAp) induced de novo secretion of the anti-inflammatory cytokine IL-13 by MSCs, with OCP exhibiting the most pronounced effect. This suggests an inherent capacity of these biomaterials to promote an immunomodulatory, pro-regenerative MSC phenotype. However, under pro-inflammatory pressure (LPS+), this response diverged significantly: DCPD maintained its ability to upregulate IL-13, OCP had a neutral effect, while HAp suppressed its secretion. Given that MSC-derived IL-13 is a potent driver of macrophage polarization towards the anti-inflammatory M2 phenotype and a suppressor of pro-inflammatory mediators [69,70], the material-dependent modulation of MSC paracrine signaling becomes a critical factor. In an inflamed in vivo setting, as induced by CFA, the favorable MSC modulation by DCPD could contribute to mitigating excessive inflammation and promoting a healing microenvironment, aligning with our observation of its superior biocompatibility and angiogenic potential. Conversely, the inability of HAp to support a compensatory anti-inflammatory response from MSCs may partly explain its association with exacerbated fibrosis and signs of rejection. Thus, the efficacy of a bone graft material depends not only on its direct interaction with immune cells but also on its ability to favorably influence the resident stromal cell compartment, which serves as a key regulator of the immune–osteogenic cascade, especially under inflammatory duress.

Our investigation demonstrated that in the absence of inflammation, calcium phosphate materials do not cause a significant influence on immune cell reactions in vitro. Furthermore, CPs do not exert a substantial influence on acute postoperative inflammation phase in healthy animals: no statistically significant differences in the number of immune cells in the peri-implant tissues were found between groups implanted with different CPs and the control group. The formation of mild fibrous tissue in healthy animals (CFA−) over the short 28-day implantation period is a natural and expected process.

The process of bone tissue regeneration is typically divided into four consecutive phases: inflammatory, soft callus formation, hard callus formation, and remodeling [72]. Our work demonstrated the first, inflammatory phase, which involves hematoma formation at the fracture site and initiation of the primary immune response. It is worth noting that the osteogenic properties of a material in subcutaneous implantation generally manifest at later time points—typically 3–6 months after the start of the experiment [73].

However, under conditions of induced inflammation, the immune response to the implanted material becomes pronounced. Monocytes and macrophages begin to actively express pro-inflammatory cytokines, leading to a restructuring of defect repair mechanisms: the balance shifts from adaptive immunity towards innate immunity. By day 10, an increase in the number of granulocytes and monocytes is observed, accompanied by a simultaneous decrease in the proportion of lymphocytes. These changes promote the formation of pronounced fibrous tissue, and among all the materials studied, hydroxyapatite (HAp) demonstrated the worst outcomes in this context.

The obtained data indicate that the application of calcium phosphate materials requires a thorough assessment of the immunological status of the peri-implantation zone, which directly depends on the overall condition of the recipient’s immune system. Consequently, underlying inflammatory processes or sensitization of the body (e.g., of allergic origin) can fundamentally alter the reaction to the implanted material. Instead of the expected biointegration, this may lead to an adverse fibrotic response or rejection, accompanied by aseptic inflammation. Such a scenario can induce autolysis of the adjacent bone tissue in the implant area, which not only blocks regeneration but also exacerbates the initial bone defect.

## 5. Conclusions

This study conclusively demonstrates that the host’s immune status is a decisive factor determining the biocompatibility and functional outcome of calcium phosphate-based bone grafts.

Our integrated in vitro and in vivo approach reveals a critical paradigm: materials exhibiting exemplary biocompatibility in a healthy physiological context can elicit profoundly adverse reactions under inflammatory conditions.

The three biomimetic CP materials, while chemically similar, displayed distinct immunomodulatory profiles in an inflamed microenvironment. DCPD emerged as the most favorable material, maintaining biocompatibility and promoting angiogenesis despite the inflammatory setting. OCP, while pro-angiogenic, also stimulated substantial fibrosis. HAp, conversely, demonstrated poor biocompatibility, exacerbating inflammation and showing clear signs of rejection.

The key finding is that the classical paradigm of biomaterial testing solely in healthy models is insufficient and potentially misleading for clinical translation. Inflammatory status must be considered a primary design criterion. We propose that future development and pre-clinical evaluation of bone graft materials should incorporate immunocompromised or disease-specific models to ensure predictive accuracy and patient safety. This work establishes a foundation for screening and selecting biomaterials tailored for patients with underlying inflammatory conditions, ultimately aiming to improve the success rates of bone regeneration therapies in diverse patient populations.

## Data Availability

All data generated in this study are available in the main article.

## Abbreviations

The following abbreviations are used in this manuscript:

ANOVA	Analysis of Variance
BMP-2	Bone Morphogenetic Protein 2
CD	Cluster of Differentiation
CFA	Complete Freund’s Adjuvant
CoA	Concanavalin A
CP	Calcium Phosphate
CT-an	Computed Tomography analysis
DAMP	Damage-Associated Molecular Pattern
DCPD	Dicalcium Phosphate Dihydrate
DCFH-DA	2′,7′-Dichlorodihydrofluorescein Diacetate
EDTA	Ethylenediaminetetraacetic Acid
ELISA	Enzyme-Linked Immunosorbent Assay
FBS	Fetal Bovine Serum
FTIR	Fourier-Transform Infrared Spectroscopy
HAp	Hydroxyapatite
HMDS	Hexamethyldisilazane
IACUC	Institutional Animal Care and Use Committee
IL	Interleukin
LPS	Lipopolysaccharide
M-CSF	Macrophage Colony-Stimulating Factor
MSC	Mesenchymal Stromal Cell
NF-κB	Nuclear Factor Kappa-Light-Chain-Enhancer of Activated B Cells
NLR	NOD-like Receptor
NO	Nitric Oxide
OCP	Octacalcium Phosphate
OPG	Osteoprotegerin
PAMP	Pathogen-Associated Molecular Pattern
PBS	Phosphate-Buffered Saline
RANK	Receptor Activator of Nuclear Factor κB
RANKL	Receptor Activator of Nuclear Factor κB Ligand
ROS	Reactive Oxygen Species
SEM	Scanning Electron Microscopy
SPF	Specific Pathogen Free
TCP	Tricalcium Phosphate
TGF-β	Transforming Growth Factor Beta
TLR	Toll-like Receptor
TNF-α	Tumor Necrosis Factor Alpha
VEGF	Vascular Endothelial Growth Factor
XRD	X-Ray Diffraction

## Appendix A

**Table A1:** Experimental groups, doses, and schedule of Complete Freund’s Adjuvant administration and biological sample collection for analysis.

Group	Agent/ Doses	Sample/ Group	Scheme	Animal Number
1	100 µL 0.9% NaCl	Native control	1st day—0.9% NaCl injection (0.5 mg/kg); hematological analysis on the 7th, 17th, and 28th day; humane euthanasia and histological analysis.	6 (1–6)
2	100 µL 0.9% NaCl	DPCD	1st day—0.9% NaCl injection (0.5 mg/kg); hematological analysis on the 7th day; subcutaneous implantation of a DPCD sample (400 mg); hematological analysis on day 17; hematological analysis and humane euthanasia on day 28 of the experiment; histological analysis.	5 (17–21)
3	100 µL 0.9% NaCl	OCP	1st day—0.9% NaCl injection (0.5 mg/kg); hematological analysis on the 7th day; subcutaneous implantation of an OCP sample (400 mg); hematological analysis on day 17; hematological analysis and humane euthanasia on day 28 of the experiment; histological analysis.	5 (22–26)
4	100 µL 0.9% NaCl	HAp	1st day—0.9% NaCl injection (0.5 mg/kg); hematological analysis on the 7th day; subcutaneous implantation of a HAp sample (400 mg); hematological analysis on day 17; hematological analysis and humane euthanasia on day 28 of the experiment; histological analysis.	5 (27–31)
5	100 µL CFA/0.5 mg/mL	only CFA+	1st day—CFA injection (0.5 mg/kg); 7 days of expectation; hematological analysis on the 7th, 17th, and 28th day; humane euthanasia and histological analysis.	5 (52–57)
6	100 µL CFA/0.5 mg/mL	CFA+ DPCD	1st day—CFA injection (0.5 mg/kg); 7 days of expectation; hematological analysis on the 7th day; subcutaneous implantation of a DPCD sample (400 mg); hematological analysis on day 17; hematological analysis and humane euthanasia on day 28 of the experiment; histological analysis.	6 (68–72)
7	100 µL CFA/0.5 mg/mL	CFA+ OCP	1st day—CFA injection (0.5 mg/kg); 7 days of expectation; hematological analysis on the 7th day; subcutaneous implantation of an OCP sample (400 mg); hematological analysis on day 17; hematological analysis and humane euthanasia on day 28 of the experiment; histological analysis.	5 (73–77)
8	100 µL CFA/0.5 mg/mL	CFA+ HAp	1st day—CFA injection (0.5 mg/kg); 7 days of expectation; hematological analysis on the 7th day; subcutaneous implantation of a HAp sample (400 mg); hematological analysis on day 17; hematological analysis and humane euthanasia on day 28 of the experiment; histological analysis.	5 (78–82)

**Table A2:** Characteristics of cylindrical calcium phosphate (CP) samples: mass and specific surface area. The surface area was measured by the BET nitrogen adsorption method using a Tristar II analyzer (Micromeritics Instrument Corp., Norcross, GA, USA). Data are presented as mean ± SE (Student *t*-test, *p* < 0.95).

Calcium Phosphate	Mass, g	Surface Area, m^2^/g
DCPD	0.6245 ± 0.0087	4.9 ± 0.2
OCP	0.5207 ± 0.0093	9.9 ± 0.1
HAp	0.4385 ± 0.0074	26.4 ± 0.4
